# Assessing the feasibility, acceptability and accessibility of a peer-delivered intervention to reduce harm and improve the well-being of people who experience homelessness with problem substance use: the SHARPS study

**DOI:** 10.1186/s12954-021-00582-5

**Published:** 2022-02-04

**Authors:** Tessa Parkes, Catriona Matheson, Hannah Carver, Rebecca Foster, John Budd, Dave Liddell, Jason Wallace, Bernie Pauly, Maria Fotopoulou, Adam Burley, Isobel Anderson, Tracey Price, Joe Schofield, Graeme MacLennan

**Affiliations:** 1 Salvation Army Centre for Addiction Services and Research, Faculty of Social Sciences, University of Stirling, Stirling, UK; 2 Faculty of Social Sciences, University of Stirling, Stirling, UK; 3 Faculty of Medicine, University of Edinburgh, Edinburgh, UK; 4 Scottish Drugs Forum, Glasgow, UK; 5 The Canadian Institute for Substance Use Research, University of Victoria, Victoria, Canada; 6 NHS Lothian, Edinburgh, UK; 7 The Centre for Healthcare Randomised Trials (CHaRT), University of Aberdeen, Aberdeen, UK

**Keywords:** Homelessness, Substance use, Drugs, Alcohol, Feasibility, Peer support, Harm reduction, Mixed methods, Intervention

## Abstract

**Background:**

For people experiencing homelessness and problem substance use, access to appropriate services can be challenging. There is evidence that the development of trusting relationships with non-judgemental staff can facilitate service engagement. Peer-delivered approaches show particular promise, but the evidence base is still developing.

**Methods:**

The study used mixed methods to assess the feasibility, acceptability and accessibility of a peer-delivered, relational intervention to reduce harms and improve health/well-being, quality of life and social functioning, for people experiencing homelessness and problem substance use. Four Peer Navigators were employed to support individuals (*n* = 68 total, intervention participants). They were based in outreach services and hostels in Scotland and England. Qualitative interviews were conducted with intervention participants, Peer Navigators and staff in services, and observations were conducted in all settings. Quantitative outcomes relating to participants’ substance use, physical and mental health, and quality of the Peer Navigator relationship, were measured via a ‘holistic health check’ with six questionnaires completed at two time-points.

**Results:**

The intervention was found to be acceptable to, and feasible and accessible for, participants, Peer Navigators, and service staff. Participants reported improvements to service engagement, and feeling more equipped to access services independently. The lived experience of the Peer Navigators was highlighted as particularly helpful, enabling trusting, authentic, and meaningful relationships to be developed. Some challenges were experienced in relation to the ‘fit’ of the intervention within some settings. Among participants there were reductions in drug use and risky injecting practices. There were increases in the number of participants receiving opioid substitution therapy. Overall, the intervention was positively received, with collective recognition that the intervention was unique and highly valuable. While most of the measures chosen for the holistic health check were found to be suitable for this population, they should be streamlined to avoid duplication and participant burden.

**Conclusions:**

The study established that a peer-delivered, relational harm reduction intervention is acceptable to, and feasible and accessible for, people experiencing homelessness and problem substance use. While the study was not outcomes-focused, participants did experience a range of positive outcomes. A full randomised controlled trial is now required to assess intervention effectiveness.

***Trial registration*:**

Study registered with ISRCTN: 15900054.

**Supplementary Information:**

The online version contains supplementary material available at 10.1186/s12954-021-00582-5.

## Background

Homelessness is often associated with poor mental and physical health and substance use [1, 2]. Poverty is one of the main reasons for people becoming homeless, but other factors, such as traumatic experiences, mental health problems, substance use, and experience with the criminal justice system, are also common [1, 3]. This study applied the European Typology of Homeless and Housing Exclusion (ETHOS) definition of homelessness to take into consideration a range of living situations including rooflessness, houselessness, and insecure or inadequate housing [4]. For people experiencing homelessness, access to support services can be difficult and accessing healthcare can be particularly challenging due to stigma, negative attitudes from staff, and inflexible services [5–8]. While many people who experience homelessness are registered with primary healthcare, they are not always able to access it when needed and frequently call on emergency healthcare services to meet their needs [2, 9–11]. Engagement can also be challenging due to problems with transportation and not being able to prioritise healthcare given other day-to-day challenges, resulting in missed appointments [5, 7, 12]. Recent evidence suggests that those who miss primary healthcare appointments are more likely to have mental health problems, including problem drug and/or alcohol use, and eight times more likely to die prematurely, than those who attend appointments [13]. Consistent access to services, regular attendance at appointments, and the ability to follow plans/regimes, may be difficult to maintain with unstable living conditions [14]. For those experiencing both homelessness and problem drug and/or alcohol use, access to, and engagement with, services can be additionally challenging [15].

Homelessness settings in the United Kingdom (UK) are becoming more psychologically and trauma informed, with many services now embedding a psychologically informed environments (PIEs) approach [16]. This provides a way of understanding how people think, feel and behave, based on their past/current experiences and environmental factors, and can inform the design and delivery of appropriate services [16, 17]. PIEs involves five key areas: developing greater psychological awareness of people’s needs; valuing training and support for all staff, volunteers and clients; promoting a culture of learning and enquiry, including in service evaluation and improvement; enabling ‘spaces of opportunity’ which seek to create effective service environments; and a focus on the rules, roles and responsiveness of the service which focuses on managing and improving relationships [18]. There is some evidence that PIEs-informed services can lead to improvements in: client mental health and well-being, housing and behavioural outcomes; engagement with health, substance use, and other care services; and reduced involvement with criminal justice and emergency services [19–22]. Prior to the study reported here, there has been a lack of application of PIEs to the field of substance use which is a notable research gap.

Harm reduction is an evidence-based approach to meeting needs and helping people to be safer [23–25]. It embodies a non-judgemental response to substance use and aims to meet people ‘where they are at’ [26, 27]. For those who are homeless and using substances, approaches include: overdose awareness training and naloxone; safe supplies of injecting equipment; drug consumption rooms; and non-abstinence based housing [26, 28]. Services which embody a harm reduction ethos provide opportunities for developing trusting and reliable relationships between staff and clients, and can enable clients to feel accepted and develop self-worth [6, 27, 29–35].

The involvement of peers, those with lived experience of particular phenomena such as homelessness and/or problem substance use, is an important aspect of harm reduction [26]. Peer-delivered services involve those with lived experience explicitly drawing on their experiences and knowledge to support those in similar situations [36–39]. Within substance use services, peers are involved in a range of harm reduction activities, including: provision of information about drug quality and safer injecting practices; injecting equipment provision and outreach programmes; provision of take-home naloxone; and a range of advocacy activities [40–56]. There are evidenced benefits to peer-delivered services for both clients and peer workers. Clients experience increased access to/engagement with services, reduced substance use and related harms, and improved housing, and report developing positive relationships and feeling ‘safe’ [36, 57, 58]. There is, however, a lack of evidence regarding the effectiveness of peer-delivered approaches using randomised designs, particularly for those experiencing both problem substance use and homelessness, and more research is therefore needed [36]. For peer workers such work can improve confidence and self-esteem, provide a sense of purpose and belonging, and support recovery [36, 59–61]. Peer workers also experience challenges in their roles, including: power imbalances; stress; and tensions over personal/professional boundaries. Peers also face the challenge of continually navigating dual identities of ‘peer’ and ‘professional’ [62]. A recent review highlighted themes of vulnerability, authenticity, boundaries, stigma, and being valued [36], with recommendations reinforcing the need for effective training, supportive and reflective supervision and management, clear role descriptions, and pay commensurate with experience/training [36, 54, 59, 63–67]. Peer support appears to be important within a PIEs framework, but there is limited evidence regarding the effectiveness of combining the two [21, 68].

There has been no study assessing the feasibility, acceptability and accessibility of a peer-delivered intervention specifically focusing on problem substance use and homelessness, alongside adopting PIEs. The Supporting Harm Reduction through Peer Support (SHARPS) study combined evidence on harm reduction, peer-delivered interventions, and PIEs, to develop a relational, peer-delivered intervention for people experiencing homelessness and problem substance use using Peer Navigators (PNs) who supported participants to address a range of health/social issues on their own terms.

## Methods

### Research questions and design

The intervention aimed to reduce harms and improve the health, well-being, quality of life, and social functioning, of people experiencing homelessness with problem substance use. The study was commissioned in 2017 by the UK’s National Institute for Health Research (NIHR) which requires a full monograph to be published at study completion. The study had a number of research questions which are addressed in full in a related monograph [69] and be available (open access) via dual publication alongside this paper, as is usual with NIHR-funded studies. This paper reports data specifically related to two of our research questions concerned with feasibility, acceptability and accessibility:

1. is a peer-delivered, relational harm reduction approach acceptable to, and feasible and accessible for, people who are homeless with problem substance use in UK non-NHS (National Health Service) settings?
2. what outcome measures are most relevant and suitable to assess the effect of this intervention in a full randomised controlled trial?

Readers who wish to read the full study are directed to the monograph [69].

A two-year mixed methods study was conducted (May 2018-May 2020) with a concurrent process evaluation to inform a potential randomised controlled trial (RCT) to assess acceptability of procedures: intervention fidelity; rate of recruitment and retention of participants; sample size and potential follow-up rates; ‘fit’ with chosen settings and target population; availability and quality of data; and suitability of outcome measures. Detail regarding the study design and intervention is provided in full in the study protocol [70] and described briefly below. The study was commissioned by the NIHR in order to inform a definitive trial that is now itself being commissioned and which this paper and monograph collectively inform. For readers interested in the options for a follow on RCT please see the related monograph. Normalisation Process Theory (NPT) [71] guided the development and implementation of the intervention as well as the process evaluation. NPT comprises four components, coherence (understanding), cognitive participation (buy-in), collective action (making it work), and reflexive monitoring (on-going appraisal) [72], and more detail is provided in Additional file 1 (Application of Normalisation Process Theory for the assessment of feasibility, acceptability and accessibility).

### Study registration

This study was registered with International Standard Randomised Controlled Trial Number—ISRCTN: 15900054.

### Ethics

Ethical approval was granted by the University of Stirling’s NHS, Invasive and Clinical Research ethics committee (NICR 17/18 Paper 35) in April 2018 and by The Ethics Subgroup of the Research Coordinating Council of The Salvation Army (TSA) in June 2018 (no reference). Four subsequent submissions were made to both committees for approval for protocol changes: all were approved.

### Peer Navigators

Four PNs were recruited and employed by TSA on 18-month contracts for 30 h per week: one PN left the role early. They were paid on a ‘specialist support worker’ salary due to the complexity of the role and the additional responsibilities. All PNs had lived experience of problem substance use and/or homelessness, and all had different experiences of recovery (which we did not view as requiring abstinence), with an understanding of the importance of harm reduction. They had all previously worked in relevant social care roles, for example within residential rehabilitation and supported accommodation, and had relevant qualifications. As part of their role, PNs received training on a range of topics including harm reduction, trauma informed care, motivational interviewing, negotiating professional boundaries as peer workers, therapeutic relationships, PIEs, and naloxone administration. They received regular clinical supervision with a Consultant Clinical Psychologist (Co-Investigator AB), informal support from each other and from JW (Co-Investigator with lived experience), and support from the project management team (TP, HC, RF, CM) and service managers who were their line managers.

### Intervention

The intervention was co-produced at a full day meeting with a range of experts including members of the study team, the PNs, those working in the field, and other individuals with lived experience of homelessness and/or problem substance use. An intervention ‘guide’ was produced for the PNs with necessary information to carry out their role including practical tools, anticipated challenges, and information about the needs of specific sub-populations.

The PNs provided practical and emotional support for a period of 2–12 months with a caseload of up to 15 people (total intervention participants *n* = 68). A fund was available to the PNs to pay for travel, food, hot drinks, clothing, and ‘phone calls, according to participant needs. While separated into ‘practical’ and ‘emotional’, in reality ‘practical support’ in the form of accompaniment to appointments could also be experienced as ‘emotional support’. Table 1 provides examples.

**Table 1:** Practical and emotional support provided by Peer Navigators

**Healthcare**
Support to access healthcare services, including attending appointments to GPs, physiotherapists, dieticians, and dentists
Access to substance use treatment
Money for travel to/from appointments
Advocacy at appointments on participants’ behalf
**Housing**
Access to housing
Money to purchase household appliances
**Benefits**
Access to benefits, including phone calls and attending appointments
**Practical support**
Paying for food and hot drinks
Clothing, stamps, phone calls
Accompanying to hairdressers
**Psychosocial support**
Helping to secure volunteering and employment opportunities
Helping to connect/reconnect with family, friends and children
**Emotional support**
Listening
Being reliable and consistent

Participants received the intervention for a maximum of 12 months. For the majority, intervention completion occurred exactly 12 months after they had started, if engagement was ongoing, to ensure there was a clear beginning, middle and end to the intervention. Participants who were based in the setting where the PN left early received a 2–2.5 month intervention (described as ‘shortened’ in contrast to ‘full’). These participants completed the intervention after 2–2.5 months. Towards the end of the intervention the PNs worked actively with their participants to ensure they were well supported by other members of staff/other services post-intervention.

### Settings

The PNs were based in three outreach services for people experiencing homelessness in Scotland, and three TSA hostels (termed Lifehouses) in England. The outreach settings in Scotland were managed by TSA, Streetwork (Simon Community Scotland), and the Cyrenians (became ‘Change Grow Live’ in April 2019). In order to assess differences between intervention and non-intervention settings, two standard care settings (an outreach service in Scotland and a Lifehouse in England) were identified that were similar to intervention settings in terms of aims, funding types, staff roles, and numbers of clients. Due to space constraints, data related to standard care settings are not reported on in this paper but are provided in the associated monograph [69].

### Eligibility criteria for intervention participants

Individuals were eligible for the study if they were: > 18 years old; experiencing/at risk of homelessness (using ETHOS definition) [4]; experiencing problem substance use (defined as the use of drugs and/or alcohol in a way that had a negative effect on their lives); and able to provide informed consent. Individuals self-reported that their substance use was affecting their lives in a negative/detrimental way and the level and nature of this varied between individuals: most participants were experiencing problem substance use that was severe and had a substantial impact on their daily lives.

### Quantitative data collection

Quantitative data were collected via what we termed a ‘holistic health check’ using six standardised measures: a socio-demographics, health and housing circumstances questionnaire developed for the study (denoted as ‘demographics’ hereafter); combined Patient Health Questionnaire-9 (PHQ-9) [73] and Generalised Anxiety Disorder-7 (GAD-7) [74]; Maudsley Addiction Profile (MAP) [75]; Substance Use Recovery Evaluator (SURE) [76]; RAND Short Form-36 [77]; and the Consultation and Relational Empathy (CARE) measure [78], see Table 2. In this table we list the PHQ-9 and GAD-7 tools separately but within the study these were treated as one measure for mental health. The term holistic health check was used to try to make the process of doing the measures more acceptable to participants. The PNs worked with each of their participants on the issues that were raised during the health check process that the person wished to have support with.

**Table 2:** Measures

Tool	Purpose	Scoring
Socio-demographic, health and housing circumstance questionnaire	To understand participants’ socio-demographic characteristics, housing status/quality, general health status, education, medication use, and future service use	Questionnaire consisted of questions regarding demographics, with yes/no options, ranges and free text. No scores were generated
Patient Health Questionnaire 9 (PHQ-9) [73]	To identify symptoms of depression (measure combined with GAD-7)	PHQ-9 is a 9-item scale. Scoring can range from 0 to 27, with scores of > 10 indicating moderate depression and > 15 moderately-severe depression and > 20 severe depression
Generalised Anxiety Disorder (GAD-7) [74]	To identify symptoms of anxiety (measure combined with PHQ-9)	GAD-7 is a 7-item scale. Scoring can range from 0 to 21, with scores of > 10 indicating moderate anxiety and > 15 severe anxiety
Maudsley Addiction Profile (MAP) [75]	To identify type, levels, and risk factors relating to drug and alcohol use. Slight changes were made to make it more suitable for the study population, including adding a question on overdose and asking about use of other drugs not included (such as Novel Psychoactive Substances). Physical health domain also used	MAP is a 60 item (with 8 additional items added to section A for the SHARPS study) Scale in four domains. Each domain is scored depending on the structure, with some using free text and others Likert scales. No overall score is generated but mean/median scores across the domains were used
Substance Use Recovery Evaluator (SURE) [76]	To identify drinking and drug use, self-care, relationships, material resources, outlook on life, and importance of these factors	SURE is a 21-item scale split into three sections, only two of which are scored. Scores range from 21 to 63, with higher scores indicating greater levels of recovery capital
RAND Short Form-36 (SF-36) [77]	To identify physical and emotional health status, the effect of health on daily activities and social activities, and experiences of pain	SF-36 is a 36-item scale. Scoring ranges from 0 to 100 on each of the eight domains, with lower scores indicating poorer health
Consultation and Relational Empathy (CARE) [78]	To assess the levels of empathy within the relationship between participant and their Peer Navigator	CARE is a 10-item scale. Scoring ranges from 10 to 50, with higher scores indicating greater levels of empathy within the relationship

Data were collected at two time points: 45 participants completed at baseline (October 2018-May 2019) and 30 at follow-up (August-November 2019). The intention had been for the PNs to collect these data but unforeseen data protection issues meant that this was not possible. Two post-doctoral academic researchers (RF and HC) worked through printed copies of all questionnaires with participants. While they were careful to build rapport and help participants to feel relaxed, the PNs were also present to offer additional support/reassurance at the time, and to take up the issues raised as part of participant support plans. The CARE measure was completed without the PN present to encourage honest responses.

### Qualitative data collection

Qualitative data were collected via semi-structured interviews with participants, staff and PNs, observations in intervention settings, and PN reflective diaries. Semi-structured interviews were conducted with a sample of intervention participants at two time points (*n* = 24 Wave One, January-March 2019; *n* = 10 Wave Two, August–September 2019), with staff in intervention settings (*n* = 12), and with the PNs at four time points (June/July 2018, April 2019, June 2019 and November 2019; three for the PN who left early: June/July 2018, November 2018 and January 2019). Academic researchers from the study team conducted the staff and PN interviews (RF, HC). Peer researchers (*n* = 8) from Scottish Drugs Forum (SDF), volunteers with lived/living experience of problem substance use and trained in research methods (including specific to SHARPS), undertook interviews with a sample of intervention participants. The involvement of peers throughout the research process has increased in recent years and can ensure research is more meaningful, as well as supporting the development of peer researchers [36, 79].

The aim of the interviews with staff and PNs was to understand experiences of, and views on, the intervention from a range of perspectives, as well as collecting data on any changes in perceptions/practices during the study. Interviews with intervention participants provided insight into their experiences of the intervention. Topic guides were developed by the study team and informed by NPT (see Additional file 2, Interview topic guides), with peer researchers inputting into the intervention participant guides. All participants were provided with information about the study via a Participant Information Sheet, the content of which was also conveyed verbally, and written informed consent was sought and obtained from all participants prior to the interview. All interviews were conducted in private rooms in services and were audio recorded with permission. All participants were provided with a debrief sheet after the interview, and intervention participants were provided with a £10 voucher to thank them for their time.

The PNs kept reflective diaries for the duration of their time in post, to provide insight into their experiences and feelings. These diaries were either typed or audio-recorded then transcribed. A template was provided with suggested prompts to facilitate their reflections. While these data, and the data from observations, do not form part of our formal qualitative data collection, diaries and observation fieldnotes were analysed and key themes drawn upon to contextualise the formal interview findings.

### Quantitative data analysis

Data from the holistic health check measures were entered into a database, and pseudonymised using an alphanumeric code. Quality assurance of data entry was confirmed though a manual check of 10% of entries. Data were analysed descriptively using frequency and percent for binary and categorical data. Continuous data were described using descriptive summary statistics. At baseline the whole cohort was described and then baseline data for those that responded at follow-up was also described. Differences between baseline and follow-up are presented with 95% confidence intervals.

### Qualitative data analysis

Interviews were transcribed in full and analysed using Framework [80] in NVivo 12. Framework enabled the analysis of the six different settings as cases and straightforward within case and between case comparison and all stages were closely followed. As data was collected at different time points with both participants and PNs, analysis sought to specifically explore whether, and how, perceptions of the intervention, its challenges and benefits, changed over time. Data analysis was iterative throughout study, supported by use of NPT to identify contextual influences on implementation across the settings. The specially convened Experts by Experience group (comprising members with lived experience of homelessness and/or problem substance use) and the peer researchers were supported (RF, HC) to participate in data analysis/interpretation which acted as a form of ‘member checking’ to enhance the validity and trustworthiness of the findings [81].

### Recruitment and retention

**Fig. 1:**
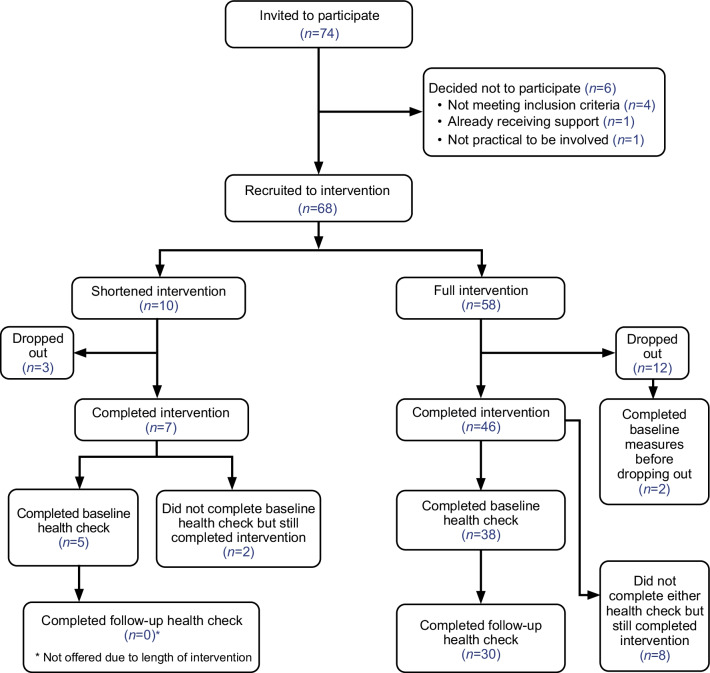
CONSORT flow diagram

Recruitment was intensive in the first two months (October, November 2018) until a desired sample size of 60–70 participants was reached, equating to approximately 19 individuals per PN (*n* = 10 for the PN who left early). Recruitment was open until mid-April 2019 to enable participants to be replaced by new participants as people withdrew in the early weeks order to maximise reach. Seventy-four individuals were invited to take part, and, of these, 68 (92%) participants were recruited. Figure 1 provides a CONSORT (Consolidated Standards of Reporting Trials) flow diagram for participant progress in/through the intervention.

Participants were able to withdraw from the intervention at any time but were not withdrawn on the basis of continued problem substance use or abstinence. Fifteen participants withdrew/dropped out: 12/68 from the full intervention (20%) and three from the shortened intervention (3/10, 30%). No withdrawals happened after recruitment ended in April 2019. In total, 46 participants completed the full intervention when it finished in November 2019.

40 of the 58 who started the full intervention completed the wave 1 health check, and 30 completed the wave 2 health check. Of the 10 participants that completed the shortened intervention, five participants provided data at baseline. While either one or two health checks/measure set were conducted with the majority of the intervention participants, there were 10 individuals in total who completed the intervention who did not complete the health check (*n* = 2 for shortened intervention; *n* = 8 for full intervention). Two participants dropped out from the full intervention and completed the baseline health check before withdrawing and their data was included in the whole cohort figure of 45 participants for our baseline measures. The research team and PNs prioritised these measures at baseline and follow-up. The reasons for not completing these were varied and included the poor mental health and challenging life circumstances of some participants. Time pressures prevented the research team from continuing to pursue these where multiple attempts had been conducted. In some instances, it was clear that participants were avoiding having these health checks/measures done and the research team therefore chose to respect their wishes, despite the importance of the measures.

## Results

In this section we first detail the intervention participant characteristics and then present our qualitative findings, detailing intervention participant, staff and PN views of the intervention. The quantitative results are then described. The order reflects our research questions concerned with feasibility, acceptability and accessibility of the intervention first and foremost, and then whether the outcome measures were appropriate. This study was not focused on participant outcomes as this would be addressed in a next stage RCT. Instead, we provide a discussion of the relevance and suitability of measures to address our second research question.

### Participant Characteristics

Participant characteristics of those who took part in the quantitative data collection at baseline and follow-up are shown in Table 3 and presented as cohorts.

**Table 3:** Pre-intervention demographics for whole cohort and participants that completed both baseline and follow-up measurements

Variable	Whole cohort		Baseline for both measures cohort	
	*N* = 45		*N* = 30	
Age years—mean (SD)	38.6 (8.7)		38.2 (8.0)	

	*n*	%	*n*	%
Male	35	78	25	83
**Marital status**				
Single	37	82	24	80
Divorced	3	7	2	7
In relationship	4	9	3	10
Widowed	1	2	1	3
White ethnicity	43	96	28	93
**Self-identified as having a disability**	30	67	18	60
Learning	1	2		
Long-term	10	22	5	17
Mental health	24	53	15	50
Mobility	8	18	6	20
Ever in residential or foster care?	20	44	14	47
**Where currently sleeping?**				
Rough	2	4	2	7
Hostel	12	27	3	10
Supported accommodation	14	31	11	37
B&B	2	4	2	7
Temp/friends/family	2	4	2	7
Own home	9	20	6	20
Other	4	9	4	13
Ever convicted of a crime?	40	89	28	93
**Number of convictions**				
1–5	10	22	8	27
6–10	8	18	4	13
11–15	4	9	2	7
16–20	3	7	2	7
> 20	15	33	12	40
Ever in prison	36	80	25	83
**Years in prison**				
Median [25th, 75th centile]	2.5 [0.6, 7]		3 [0.8, 7]	
**Self-rated health in last 6 months**				
Very good				
Good	4	9	4	13
Fair	18	40	10	33
Bad	15	33	10	33
Very bad	7	16	6	20
Missing	1	2		
**Reason for taking non-prescribed medication**				
Physical health	3	7	3	10
Mental health	5	11	4	13
Both	6	13	5	17
Taking prescribed medication for a drug or alcohol problem	28	62	17	59
Ever spent time as hospital inpatient	43	96	29	100
Ever thought about self-harm or suicide	35	78	21	72
**Ever acted upon thoughts**				
No	10	22	9	31
Yes	21	47	12	41
Prefer not to say	5	11	4	14
Drug overdose in the past month	2	5	2	7
Ever in drug treatment	32	73	24	80
Number of times in drug treatment	*n* = 32		*n* = 24	
Median [25th, 75th centile]	2.5 [1, 5]		2 [1, 4]	
Time in last treatment (months)	*n = 28*		*n* = 21	
Median [25th, 75th centile]	10.5 [4.5,54]		12 [6, 24]	

The mean age of the whole cohort was 39 years and 35 (78%) were male. The majority (96%) of participants described their ethnicity as ‘white’. Twenty-two (49%) described their health as being ‘fair’ or ‘good’ in the past 6 months. Twenty participants (44%) had been in residential or foster care in their lives. Almost all participants (96%) had been in hospital as an inpatient at some point in their life. Thirty-five participants (78%) reported having ever thought about self-harm or suicide, and 51% in the previous month. Two had overdosed in the last month. Thirty-two (73%) had ever been in drug treatment. Participants that responded at both time-points had broadly similar demographic characteristics.

### Qualitative results

Qualitative interviews were analysed using NPT as a guiding framework. Analysis was designed to explore how individuals understood, adopted, or perceived the intervention, how participants engaged with the intervention, how staff experienced hosting the intervention, how the PNs made sense of their role, and other contextual factors impacting delivery. Additional file 1 details the application of NPT to the assessment of feasibility, acceptability and accessibility. Due to space limitations, we report the full NPT analysis in our related monograph [69]. In this paper we present qualitative data to address the first research question: is a peer-delivered, relational harm reduction approach acceptable to, and feasible and accessible for, people experiencing homelessness with problem substance use in non-NHS settings? The PNs are described below as ‘PN A-D’, Scottish dialect within quotations has been clarified where necessary, and gender pronouns have been removed and replaced in the quotations by ‘they/they’ve’ in brackets to anonymise.

### Intervention participants

Participants reported valuing the PNs and benefitting from the support provided. Some indicated that they required support to attend appointments, in many cases not due to a lack of capacity to engage but because they had experienced significant stigma in previous interactions with services. Some expressed that the PNs helped to reduce the anxiety involved in attending appointments by using a combination of empathy and humour:

To be honest (they’ve) made it a lot easier for me to get to appointments, like at the hospital… I was getting down on it, no [not] wanting to go because I remember like last year, it was just like a big long journey on the bus, ken [you know] like being on your own and that, but we go through and have a laugh*.*

Some participants found the welfare/benefits system difficult to navigate, leading to situations where many had given up applying for support. Others noted the stress involved in attending welfare/benefits appointments and, in these situations, the PNs used skills such as providing reassurance and distracting to reduce stress and encourage more positive experiences:

Aye [yes] my PIP [Personal Independence Payment] and ESA [Employment Support Allowance]. I was around the bend with them mate, but (they) took me, there is a boy in [city/town] who does all the PIP forms so [PN B] came with me to that appointment, met me at the train station, came and sat with me and all that. And spoke to the woman because I cannae [can’t] to be honest.

Participants reported a number of positive outcomes which helped them to continue to value the intervention, including greater access to, and engagement with, healthcare, support services, and housing support. The support offered by the PNs was described as the key factor that led to these positive outcomes. The PN’s presence helped overcome stigma when accessing services, with participants feeling empowered to advocate for their own needs due to their steadying presence:

That’s how it’s helped me because now, since I’ve been working with [PN C], I’ve started literally thinking, ‘no I am not going to ask them to do it, I’ve got to do it myself’.

This empowerment often laid the foundations for developing independence further. While some participants attributed such progress to engagement with the PNs, the PNs would encourage them to recognise their progress as a product of their own actions:

Most of my progress has been because of [PN B]… They would say it was because of me… because (they) don’t want to take credit for it. But it is, I wouldn’t have engaged the way I have if it hadn’t been for [PN B]. (They) motivate you and get you thinking positive.

All participants interviewed during the follow-up interviews reported positive changes as a result of the PN intervention. For some, these changes related to harm reduction and substance use stability:

Because now I’ve got myself stable, on a script and that.

I’ve cut down on my drug intake, but I’ve still had a couple of relapses.

I’ve got myself on a prescription now and stopped using because I am coming down on my methadone as well because I am going into rehab.

Many participants also described less tangible but nevertheless important changes, including increased confidence and hope. This appeared to stem from the respect shown by the PNs who acted as positive role models:

Like (they) urge me to go and do what I’ve got to do, (they) were an alcoholic and on drugs at one point of (their) life ken [you know] but look at (them) (they’ve) got a partner now and a car, and a job, and (they’re) somebody to look up to basically, (they’re) a good role model.

The lived experience of the PNs was highlighted by participants as being particularly helpful, enabling trusting, authentic, and meaningful relationships to be developed and, in turn, supporting participants to make positive changes to their lives. Participants’ awareness that PNs had first-hand experience of challenges, similar to their own, helped to reduce barriers to trust, enabling them to be more comfortable sharing difficulties, including honesty about drug and alcohol use:

I can be honest, open, and tell (them) exactly how it is.

I get a lot of support, a lot of talk, like if I feel that I am going to relapse.

I can go and talk because (they’ve) been there in the past.

Once trust developed, many noted the value of the PN’s ability to give honest advice and engage in ‘straight-talking’:

(They) wouldn’t beat about the bush. (They) wouldn’t say’ ‘oh come here poor little lad/lass’. (They’d) sort your head out, in a good way.

(They) told me straight… ‘listen if you are happy with your lifestyle live with it, if you are not, do something about it’.

In most interviews, descriptions of ‘s/he just gets it’ linked to the PN’s lived experience, with the knowledge that they too had been there and therefore ‘understood’:

(They’ve) had (their) own life experiences, they’ve had (their) own journey, and whatever journey (they’ve) been on in (their) life it’s helping (them) to work in a more constructive manner. Nothing against the professionals that I’ve worked with in the past but, due to the fact that (they’ve) been there, I think that makes it so much easier to open up with. And (they) really do try to make you feel as comfortable and at ease as you could be.

The shared experience, combined with a commitment to harm reduction and to be non-judgemental alongside other values, helped participants to feel accepted:

[PN D] understands that although I am a recovering alcoholic I will sometimes still go out and have a drink when I am a bit low in morale and it doesn’t bother (them). As long as I don’t show up absolutely wasted they’re absolutely fine. (They’re) the best worker I’ve ever had in my life….

Finally, although between staff conflict did not feature in all settings, and was not referenced in all participant accounts, this emerged in some of the interviews, as well as in the reflective diaries and conversations between the PN and project management team. This finding suggests elements of stigma and disempowerment, with participants comparing the PNs to other staff:

I shouldn’t really say this but (they’re) having a bit of trouble with the staff here… they don’t want to help you, do you know what I mean? Not at all.

The shared experience between participants and the PNs enabled trusting relationships to be developed which helped them to ‘buy into’ the intervention and, in turn, equipped them with the confidence to make positive changes to their lives.

#### Intervention setting staff

Challenges were experienced in relation to the ‘fit’ of the intervention in some settings. Some members of staff perceived there to be a cross-over between the PN role and host organisation’s Support Worker role, despite the distinctiveness of the roles being simultaneously recognised. This was more notable in the intervention residential settings. All participants based in the residential settings had a Support Worker; if they became involved in the intervention, they also had a PN. If participants moved on from the residential setting over the course of the intervention, they were only supported by their PN at this stage. Some interviewees commented on the tension that could ensue where dual support was offered:

The biggest problem has been in that crossover between Support Worker and what [PN A’s] role is. You get them saying ‘oh well I’ve got another Support Worker’ but they will kind of taunt the other residents. Or they will say ‘well why haven’t I’? They get jealous, and envious that they think somebody else is getting something that they are not.

A more substantial frustration for some members of staff was that the PNs had the responsibility of supporting service users without the requirement to also conduct structured assessments and associated paperwork, giving them more time for informal support:

[PN A] gets them treats and all that and they are all made up when they see [PN A], do you know what I mean? And (they’ve) got that time to sit and talk to them where sometimes we haven’t.

For some interviewees there was the sense that the PN’s ability to spend time, and to undertake more enjoyable activities, positioned their role against the Support Worker role:

Sometimes the staff can feel that we are a little bit like saying no and [PN C] is always the yes. It’s like ‘good cop, bad cop’.

Due to this lack of role clarity, especially in the initial stages, some staff interviewees suggested that additional preparatory work would have allowed a greater level of contextual integration and fit:

The service users think it’s a great thing. I think some of the staff are maybe not convinced overall, just because they see some of the challenges, or the overlap. But having said that they can see the value of what (they are) doing for the service users. I think it’s a clash of roles, not what [PN C] is doing. I guess it’s how we integrate it into the service.

Staff expressed that the intervention was broadly acceptable within the services they were hosted in and was well-received by service users. Even where tensions were articulated, staff members recognised the benefit of a designated staff member being able to spend more time with participants, including outside of the service, and to spend this time in a more flexible way. Relationships among all staff, including the PNs, were generally positive. Almost all interviewed staff reflected that an important component of the intervention was the PNs’ ability to build therapeutic relationships with participants who often found it difficult to trust:

[PN D] doesn’t go to anybody and start talking to them. They get drawn to [PN D], they’ve heard about (them) and they’ve seen how (they) work. (They’re) like a magnet.

This ability was attributed to a combination of personality, training, and the experience gained from having lived experience of difficult times:

Some of the stuff with [PN C’s] skills haven’t necessarily been taught, I think (they’re) natural. In some areas (they’re) an absolute natural.

It’s been that mix of ‘I’ve been there’, so it’s the experience side of what that means in terms of being able to build relationships with somebody. But also the training they’ve got. So there is that technical understanding of what might be happening with somebody that probably has enlightened them as to where they were when they were struggling, you know, and being able to reflect on that.

There was a sense that having lived experience helped the PNs to notice important details that others may overlook:

There is a deeper understanding of the smaller detail that would have been missed by me and others because we’ve never been there.

Staff commented that the PNs had tenacity to help their participants:

(They’re) very focused on the job in hand. And it appears to me that they go over and above. You know, (they are) desperately trying to help the people that they are working with.

Finally, staff members identified that the PNs were particularly skilled at engaging with individuals who had been referred to by staff in services as ‘chaotic’ or ‘hard to reach’. The PNs appeared to be able to engage with people who had been described in this way more quickly than non-peer staff members. They expressed that the intervention was especially beneficial because the PNs were uniquely able to engage these clients:

The ones that they’ve either gravitated towards, or have gravitated to them, are the ones that are still really, really struggling with addiction and complex needs. It’s not been the ones that, you know, have been on a script for years and not touched anything else. That’s not who they’ve worked with.

By being able to build meaningful relationships, the PNs were able to work with those who had significant health issues, and minimal engagement with services.

#### The Peer Navigators

The pace of the support was considered to be an important factor that facilitated buy-in, as expressed by the PNs themselves. This ability to work at a participant’s pace, unencumbered by some of the demands and targets commonly required in social care, was viewed as crucial. It allowed participants time to build trust in the PNs, and engage as much or as little as they were able to. Connected to the data above from staff in the settings, the PNs expressed that they had more time available than a traditional Support Worker would:

My aim’s to build a relationship with the clients that we are going to be working with. Getting on with them on a personal level and not having that little countdown clock on. You do your work with them and get a really nice relationship with them. Working through that with them like at their pace.

As PNs, the main thing that we’ve got on our side is time. That is what separates me from a Support Worker is that it’s very much ‘we are in this together’ kind of relationship. We will do this. We will go and do that.

The PNs used a range of skills to respond to the needs of participants. Drawing from their training and lived experience, they were able to connect challenging behaviours to underlying trauma or other negative experiences. In some settings, the difference in approach caused some tensions, raising questions over contextual integration and coherence with existing group practices. As described in the section above, this was partly related to staff perceptions of role cross-over, but also of the need for better communication:

Every single person I am working with has got a Support Worker based in my service so there were a lot of worries about treading on toes and duplicating work and stuff which hasn’t really been the case. We are not duplicating work at all, but it’s just when everyone is working so closely together there is a massive lack of communication.

The PNs’ person-centred approach meant that the role was sometimes difficult to define and communicate. The PNs expressed that they had often explained their role to staff, but it had not always been understood. The PNs were very clear on their own roles demonstrating a high level of coherence:

My role is to go on and improve the situation that they are in by like planting little seeds but also doing all the networking I can be doing around housing, thinking of out the box for opportunities for them, or getting them to appointments, accompanying them.

A combination of confidence in their training, the support put in place, and their lived experience, enabled the PNs to embrace an approach that participants and staff alike considered unique. The role was clearly differentiated from many other third sector (not-for-profit) and statutory service roles. All PNs described the importance of slowly building a therapeutic relationship, based on trust and mutual respect. Yet, once established, the individualised approach meant that the support they provided was highly varied:

Getting people on prescriptions, taking people to specialist wound clinics that there is no way they would have attended, or even been able to attend, which could have had dire consequences for them. Child protection stuff, adult protection stuff, basic food shopping, getting power on, homeless applications to get people in housing when they are rough sleeping, specialist eating disorder clinics that people probably wouldn’t have referred themselves to, or been able to get to, mutual aid meetings, various hospital stuff, BBV [Blood Borne Virus] stuff, getting people started on Hep[atitis] C treatment that probably wouldn’t have and were very shut down to the whole idea in the beginning. House clearances and new tenancies.

In responding to individual needs, and following a PIEs approach, the PNs understood that people’s challenges were often interrelated:

It was difficult because [name of staff member] wanted me to just be working with them around the drugs and alcohol. But obviously, when people are coming into me and you are doing a whole holistic thing around all the trauma they have suffered, you are not just sat there talking about drugs and alcohol, you are talking about sexual abuse, about them working on the streets, about all the different things.

Despite the challenges experienced by participants, they were able to make positive changes to their lives, as has been noted. The PNs spoke of their pride in these achievements, showing genuine investment in their participants’ well-being:

He is much more settled now. He’s in a relationship. He’s looking a lot better. He’s put on quite a bit of weight. He’s drug-free. He enjoys attending the group session and always has a really good input to it. His confidence has improved quite a lot. And I get a sense that he’s thinking more about the future and where he might go next, as opposed to where he’s been and feeling stuck. This is just lovely to see.

He is doing amazingly well, absolutely brilliantly. He’s not using drugs at all. No heroin, no crack, and he hasn’t done for three months minus one slip up. He is currently volunteering. He wants to go into a similar type of work to what I am doing, and he’s about to start a qualification.

Given the relationships that had been developed, the team anticipated that the PNs and participants might find the conclusion of the intervention emotionally difficult. For some participants this came as a surprise, despite the parameters being clearly laid out when they were recruited to the study. The PNs described how some participants seemed ‘lost’. They also acknowledged the irony of a relational intervention where the relationship is advocated as key but is explicitly time-limited. While expressing these frustrations, they understood the need for a time-limited feasibility study to inform a subsequent trial. The end of the intervention period was carefully prepared for by both the PNs and the study team. The PNs each developed individual ‘wind down’ plans for all participants and were supported by service managers and the project management team to complete these. A debrief sheet was also available to participants when they left the intervention.

Lastly, the study involved the recruitment of four individuals with different life experiences but common to all was their experience of problem substance use and/or homelessness. All had different experiences of, and approaches to, recovery. All had unique skills, abilities and personalities. While they described their role as being challenging at times, they also felt fulfilled. When asked about their experiences of being part of the study, all shared positive experiences:

It’s been challenging but it’s been an amazing experience.

I felt quite important, I felt like I was special, yeah because this is my first proper job that’s not just like menial. So yeah getting on the train and having all that paid for, and the training stuff, felt nice, it felt good.

The PN interviews demonstrate that, with appropriate training and support, peers can be employed in demanding professional roles, use their autonomy to make complex decisions in the support of vulnerable people, hold associated responsibilities for personalised budgets and case management, and perform a diversity of tasks and processes exceptionally well. They excelled in these specialist roles, providing unique support to individuals experiencing profound challenges to reduce harms and enhance health promoting behaviours.

### Quantitative results

The quantitative results from the ‘holistic health check’ are now described by presenting baseline data from both the whole cohort and the cohort of participants that did both baseline and follow-up measures. At baseline, 43 (96%) of participants reported using at least one substance. The median [25th, 75th centiles] number of substances used was 5 [3, 5]. A range of drugs, including alcohol, were used, with opioids (which includes all opioid drugs i.e. prescribed and illicit) (*n* = 25, 57%, oral use), heroin (*n* = 22, 50%, by injection and smoking/chasing), and crack cocaine (*n* = 23, 52%, mostly smoking/chasing, some injecting) being most frequently reported. Self-reported substance use by participants in the previous 30 days is described in Table 4.

**Table 4:** Self-reported substance use at baseline and follow-up

Substance	Baseline whole cohort	Baseline for both measures cohort	Follow-up
	*N* = 44*	*N* = 30	*N* = 30
**Alcohol *n* (%)**	21 (48)	16 (53)	15 (50)
Days	12 [2, 30]	19 [2, 30]	5 [1, 30]
**Heroin *n* (%)**	22 (50)	15 (50)	9 (30)
Inject	9	5	6
Smoke	13	10	3
Days	13 [2, 30]	6 [1, 30]	2 [1, 3]
**Cocaine (crack) *n* (%)**	23 (52)	15 (50)	11 (37)
Inject	17	12	8
Smoke	5	2	2
Days	13 [2, 30]	11 [1, 21]	2 [1, 8]
**Opioids *n* (%)**	25 (57)	17 (57)	23 (77)
Oral	22	14	22
Sniff			1
Days	30 [30, 30]	30 [30, 30]	30 [30, 30]
**Benzos *n* (%)**	13 (30)	9 (30)	7 (23)
Days	30 [4, 30]	30 [4, 30]	30 [4, 30]
**Gabapentinoids *n* (%)**	15 (34)	11 (37)	7 (23)
Oral	14	9	6
Snort/sniff	1	1	1
Days	3 [2, 30]	10 [2, 30]	2 [2, 30]
**Cannabis**	19 (43)	13 (43)	13 (43)
Smoke	19	13	13
Days	15 [2, 27]	15 [14, 30]	30 [30, 30]

Cells are *n* and (%) except for rows with days which summarises median [25th, 75th centile] days of use. *One participant who completed the baseline measures did not complete the full suite of measures (2/6 completed), hence 44 rather than 45. This participant did not complete follow-up measures as they completed the shortened intervention

At follow-up there were changes in patterns of substance use. ‘Opioid use’ (which includes all opioid drugs i.e. prescribed and illicit) increased from 57% at baseline to 77%: all those who were taking opioids continued to use them, and six individuals started taking these between baseline and follow-up. Of the 15 that were taking heroin at baseline (both measures), 10 had stopped by follow-up, but four participants who were not taking heroin had started to do so. Crack cocaine use had fallen from 52% (*n* = 23, whole cohort) to 37% (*n* = 11), as had gabapentinoid use (from *n* = 15, 34% to *n* = 7, 23%). Two participants had experienced overdose in the last month at baseline (Table 5). At follow-up, no participants reported an overdose in the last month. The proportion currently engaged in opioid substitution therapy (OST) increased from 57% (*n* = 17, both measures) at baseline to 67% (*n* = 20) at follow-up; most of these were prescribed methadone with median doses of 70 ml at baseline and 60 ml at follow-up. There was a marked reduction in injecting behaviour, with 36% (*n* = 16) of the whole cohort reporting injecting in the past month at baseline and 7% (*n* = 2) at follow-up (see Table 5).

**Table 5:** OST and injecting drug use behaviour

	Baseline whole cohort	Baseline for both measures cohort	Follow-up
	*N* = 44	*N* = 30	*N* = 30
**Current OST**	25 (57)	17 (57)	20 (67)
**Current methadone patients**			
Years taking methadone	*N* = 23	*N* = 16	*N* = 17
Median* [Q1, Q3]	2 [0, 5]	1 [0, 2]	1 [0, 3]
Current dose (ml)	*N* = 23		*N* = 15
Median [Q1, Q3]	70 [50, 100]	78 [50, 98]	60 [45, 90]
**Current buprenorphine patients**			
Years taking buprenorphine	*N* = 3	*N* = 2	*N* = 3
Median (Min, Max)	1 (1, 2)	1 (1, 1)	0 (0, 1)

	*N* = 44	*N* = 30	*N* = 30
**Injected in the past month**	16 (36)	10 (33)	2 (7)
If yes, on how many days did they inject	*N* = 10	*N* = 5	*N* = 1
Median [Q1, Q3]	1.5 [1, 30]	1 [1, 3]	30
**Overdose in the last month**	2 (5)	2 (7)	0 (–)

Cells are *n* and (%) except for rows with days which summarises median [25th, 75th centile] years of use. *0 years implies less than 1 year of duration

There was overall improvement in SURE total score between baseline and follow-up (a higher score indicates recovery is progressing). At baseline the median [quartile 1, quartile 3] SURE score was 41.5 [35,48] in those that provided baseline data only. In those that provided both data points, the median was 39.5 [34,48] at baseline and 46.5 [36,54] at follow-up. There were particular improvements in substance use and self-care sub-domains, as shown in Table 6. The relationship between the participant and their PN was excellent at baseline and remained so at follow-up (Table 6).

**Table 6:** SURE and CARE scores at baseline and follow-up

	Baseline whole cohort	Baseline for both measures cohort	Follow-up
	*N* = 44	*N* = 30	*N* = 30
**Sure domain**			
Substance use	12 [10, 14]	12 [9,14]	14 [10,16]
Self-care	7 [6, 8]	7 [5, 11]	9 [6, 12]
Relationships	4 [3,6]	11 [8,12]	10 [9,12]
Material resources	11 [8.5,12]	7 [85,8]	7 [6,9]
Outlook	7 [5.5,9.5]	4 [3,6]	5 [3,8]
Total	41.5 [35,48]	39.5 [34,48]	46.5 [36,54]
**CARE**	49.5 [43, 50]	50 [43, 50]	49 [46, 50]

Cells are median and [quartile 1, quartile 3]. Higher scores are better for SURE (for total min 21 to max 63) and CARE (min 10 to max 50)

Table 7 contains summary statistics on mental health measures and MAP physical domain. The mean scores for mental health outcomes (PHQ-9, GAD-7) improved overall, and the combined score of these (PHQ-ADS) demonstrated a reduction in the severity of self-reported depression and anxiety for many. However, for some participants mental health had deteriorated at follow-up. Physical health, reported by MAP, also improved at follow-up.

**Table 7:** Baseline and follow-up PHQ-9, GAD-7, PHQ-ADS and MAP-physical domain

	Baseline for whole cohort	Baseline for both measures cohort	Follow-up
	*N* = 44	*N* = 30	*N* = 30
**PHQ-9**			
Mean (SD)	15.5 (7.3)	14.2 (7.3)	13.6 (6.5
Mean difference (95% CI)			− 0.6 (− 0.3 to 2.2)
**GAD-7**			
Mean (SD)	14.7 (6.1)	14.3 (6.3)	11.7 (6.9)
Mean difference (95% CI)			− 2.6 (− 5.4 to 0.2)
**PHQ-ADS**			
Mean (SD)	30.2 (12.6)	28.4 (11.9)	25.3 (11.7)
Mean difference (95% CI)			− 3.1 (− 7.8 to 1.7)
**MAP-physical**			
Mean (SD)	30.9 (13.4)	31.0 (12.4)	28.3 (12.7)
Mean difference (95% CI)			− 2.5 (− 6.6 to 1.5)

Mean difference is follow-up-baseline. CI confidence interval. Higher score = more severe condition

## Discussion

This paper has outlined a novel intervention to reduce harms and increase health promoting behaviours and quality of life for people with substance related problems who experience homelessness. We have presented data answering two research questions:

1. is a peer-delivered, relational harm reduction approach acceptable to, and feasible and accessible for, people who are homeless with problem substance use in UK non-NHS (National Health Service) settings?
2. what outcome measures are most relevant and suitable to assess the effect of this intervention in a full randomised controlled trial?

We take the discussion of these questions in turn and connect our findings to related literature.

### Feasibility, acceptability and accessibility of intervention

The SHARPS peer-delivered, relational harm reduction intervention, delivered in third sector (not-for-profit) residential and outreach settings, was acceptable to, and feasible and accessible for, people experiencing homelessness with problem substance use. NPT was a useful guiding framework for both the intervention and process evaluation and was well-placed to support each [71]. As Murray et al. outline, NPT recognises that healthcare is collective and requires a range of interactions from different actors. It provides a framework to help understand how these interactions shape each other and also how they can be optimised [71].

The intervention was perceived to be beneficial by the study participants, highlighting the acceptability, accessibility, and feasibility of the approach. Key benefits were the reduction in harmful behaviours, positive service engagement, and improvements in physical and mental health. A key beneficial component for participants was the PNs’ lived experience which facilitated trust and honesty and the development of effective therapeutic relationships, something mirrored in wider literature [35, 46]. Participants reported feeling a strong affinity with the PNs related to shared experiences that did not generally exist with other staff members, which is also echoed in the literature on peer support in homelessness services [82, 83] and among people who use substances [54, 58, 84]. Practical support, sometimes enabled by financial support, enabled participants to engage with services where they had previously had negative experiences, something other studies have also highlighted [5–8]. This included: OST and other substance use treatment, mutual aid groups, GPs, dentists, housing, welfare benefits, among others (see Table 1). There may have been other services participants engaged with on their own, as a result of the support provided by the PNs, that were not captured in our data. The breadth of services the PNs helped participants engage with was considerable: in a future RCT it would be important to collect data regarding how individuals were supported to engage with such a range of services.

Intervention staff generally felt that the intervention was acceptable, accessible, and feasible because of the PNs’ lived experience, flexible role and visibility, and training and skills. In some settings, staff were less positive about the intervention, with concerns about the peer element of the role, and tensions with other staff roles, which has also been noted in the literature [53, 85]. Some staff seemed unconvinced of the need to give such intensive support to the people that the intervention was targeting. This highlights the need to provide more information to all staff in service settings regarding why it is important to provide such intensive support to this group of people, and utilise peers in service delivery. In some settings staff had not had the benefit of training on PIEs, trauma informed care, and the reasons why individuals end up experiencing homelessness and problem substance use which might be a factor here. Some tensions were also reported where PNs were regarded with some degree of suspicion because of the flexibility of their roles and some misunderstandings regarding the role. While the PNs fully understood how their roles were different from Support Worker roles, this was not the case for some staff. These observed tensions may reflect resource challenges experienced in health/social care environments, and the particular challenges of ‘key-working’ in residential settings [86]. In addition, this finding relates to challenges experienced by peers in general [58, 87–89], and within the sphere of problem substance use and homelessness most specifically [36, 60, 61]. Indeed, findings also indicated the presence of stigma towards histories of substance use that the PNs had to actively manage as part of their roles. Problem drug use is highly stigmatised [90], and this can be compounded by the experience of other, often related, experiences including homelessness [5, 14, 91–93].

The intervention was feasible, acceptable and accessible to the PNs themselves because of the following key features: flexibility of role, actively valuing their lived experience, receiving extensive and varied training, and receiving diverse, responsive and ‘layered’ support (different types of informal/formal support from study team and service managers). Caseloads for the full intervention were experienced as high overall, but the flexibility of the role allowed the PNs to respond nimbly, providing more or less intensive support to individuals at any given time to manage this. This was experienced as following a PIEs approach, as well as fitting with an emerging evidence base which highlights the importance of flexible and person-centred support for those that are severely and multiply disadvantaged [94]. The PNs were proud of their role and the achievements of participants. While they identified tensions between their roles and those of other staff in their host services, they generally attributed these to lack of understanding of the PN role. The training received was vital in helping them to develop in confidence, knowledge, skills, and understanding. This is in keeping with best practice in working with peers, as identified by Miler et al. [36].

### Quantitative ‘holistic health check’ measures

#### Overview

We now turn to answering the second research question this paper posed: what outcome measures are most relevant and suitable to assess the effect of this intervention in a full randomised controlled trial? Quantitative data using the holistic health check suite of outcome measures succeeded in gaining an in-depth understanding of the participant population. Many identified as having a disability, had previously been convicted of criminal offences, had been in prison, and had been in local authority care as a child, echoing experiences described in wider literature [92]. Many felt that their physical health was fair or poor, and reported a range of physical and mental health problems. Participants also reported using a range of substances and had previously experienced substance use treatment. These data highlight the complexity of participants’ lives, and the wide range of problems they experienced, suggesting that the PNs were able to engage with participants who met the study inclusion criteria, for whom engagement with previous services had been problematic.

#### Suitability for use

Six measures were used in the holistic health check (demographics, PHQ-9/GAD-7; MAP; SURE; RAND SF-36; and the CARE measure). Of these, the first was compiled by the project management team and the other five were validated tools. The demographics measure was based on other measures used elsewhere, or in previous work by the team [29, 95, 96], and included health conditions and service use. Testing the suitability of several measures led to inevitable duplication which led to some participant frustration because of perceived repeated questions of the same nature, and the increase in time taken to complete all the questionnaires. Because of this, the total completion time was most likely too long for a single sitting. The time range for completion of all questionnaires was 30 minutes-two hours. Researchers felt 30 min was optimal, although this relied on participants answering the questions concisely and themselves wanting to complete the questionnaires fairly quickly. Researchers planning RCTs should choose outcome measures carefully to avoid duplication and reduce completion time.

We used the RAND SF-36 measure to capture generic quality of life, an essential outcome to capture in most RCTs. The RAND SF-36, however, was unpopular. The American language and expressions made it less suitable for the UK context of the research. Some of the questions were not relevant to the client group or sensitive to their circumstances; for example, references to the ability to undertake moderate activities such as ‘bowling or playing golf’. We conclude that the RAND SF-36 is therefore not suitable for measuring generic health for this client group. A better choice would have been the EQ-5D, which comprises five generic and plain language questions and has been used successfully with this client group [97]. It can also be used in health economic evaluations.

The measures we used for mental health, PHQ-9 and GAD-7, captured the poor mental health of this cohort at baseline. We recommend their use in RCTs with this client group for several reasons. Both measures were easy to use, with no missing items recorded, and neither measure exhibits ceiling/or floor effects with this client group. The outcome measures are sensitive to change and, in this study, at the cohort level, there was a change score signal in the desired direction. 

Use of substances is an essential outcome to measure for RCTs evaluating PN interventions that focus on reducing harms from such substances and should therefore be collected. We collected detailed data on drug use via the MAP, and aggregated data on numbers of days drugs (any) and alcohol were used using the SURE measure. Using either is acceptable but using both is not required. There are trade-offs to make when choosing one: the SURE is quicker and less onerous but does not allow for assessment of risk as the MAP does. However, we do not recommend use of substances as a single primary outcome for a next stage trial on PN interventions. Because the SHARPS PN intervention addressed the complex and mutually reinforcing nature of tri-morbidity experienced by participants by targeting the underlying and under-recognised mental health distress experienced, we suggest using a mental health outcome measure such as PHQ-9 and/or GAD-7 as co-primary outcome.

The CARE measure recorded patient feedback on the PN; this was well received and quick to complete. We recommend collection of this measure in RCTs evaluating PN interventions given therapeutic alliance can be a mediator of intervention effects.

### Strengths and limitations of the study

Qualitative data were very rich and insightful, providing insight into, and interrogating, feasibility, acceptability and accessibility from different perspectives. The quality of the interviews conducted by the peer researchers was good with some learning as the study developed. For example, on reviewing transcripts the team recognised there was a lack of prompting in the Wave One interviews, which was addressed at Wave Two. The organisation and co-ordination of both waves across multiple sites required considerable involvement from academic researchers, our SDF partners, and the PNs. The costs and benefits of this approach would require consideration in an RCT. There was substantial attrition in this study which, given the nature of the participant group, was to be expected in light of other literature with participants experiencing homelessness [98]. In a future definitive trial this will need to be addressed via strategies deliberately employed to maximise participant retention through the life of the study [29, 99], especially for control participants. An RCT evaluating this intervention should systematically record participant engagement throughout the study, as well as building in such retention strategies.

One limitation is that those who withdrew from the study were not interviewed. In terms of completeness of data (see Fig. 1), ten individuals who completed the intervention declined to complete the health check (measures), despite completing the intervention whether shortened or full. This was despite prioritising these activities and multiple attempts by the study team and PNs to complete them. The reasons for non-completion varied (as detailed earlier) and related to an individual’s circumstances. The PNs and study team were responsive to these and agreed on an individual basis when to stop pursuing completion. In an RCT it would be essential for all to complete the baseline data collection in order to be entered into the trial. Another limitation, with important implications for future research and embedding PNs into practice, was that frontline staff were not provided with enough information about the value of peers and the role of the PNs, prior to the study commencing. Tensions between staff and the PNs were observed and these could have been minimised or eliminated if sufficient information was provided to staff, ensuring clarity on the different roles.

## Conclusion

This study has established that a peer-delivered, relational harm reduction intervention is acceptable to, and feasible and accessible for, people experiencing homelessness and problem substance use. The qualitative findings highlighted the positive outcomes reported by intervention participants, which both they, and staff, attributed to working with PNs with relevant lived experience, and having time to spend with individuals. Challenges were reported, particularly in terms of tensions between PNs and other staff, with key lessons for how peer support is delivered in practice. While the study was not outcomes focused, participants did experience a range of positive outcomes. Quantitative data collection using the holistic health check suite of outcome measures succeeded in gaining an in-depth understanding of the participant population. While they were largely suitable for people experiencing homelessness and problem substance use they should be streamlined to be less burdensome. Two primary outcomes, that of substance use and mental health, are recommended in a next stage trial. A full RCT is now required to assess intervention effectiveness.

## Supplementary Information

**Additional file 1.** Application of Normalisation Process Theory for the assessment of feasibility, acceptability and accessibility.

**Additional file 2.** Interview topic guides.

## Data Availability

Due to the sample size and known geographical locations, there is a risk that individuals may be identified if the datasets were made available. As the interview transcripts contain a considerable amount of contextual data, it may be possible to identify participants, including the members of staff who were interviewed. This study involved important partnerships with a range of organisations with whom the study team have developed trusting working relationships, with the expectation that any arising sensitivities would be carefully considered. For these reasons, the qualitative and quantitative data sets are not available for sharing.

## Abbreviations

BBV: Blood borne virus
CARE: Consultation and Relational Empathy
CI: Confidence interval
CONSORT: Consolidated Standards of Reporting Trials
EQ-5D: Euro-QoL-5 Dimensions
ESA: Employment support allowance
ETHOS: European Typology of Homelessness and Housing Exclusion
GAD-7: Generalised Anxiety Disorder-7
MAP: Maudsley Addiction Profile
NHS: National Health Service
NPT: Normalisation Process Theory
OST: Opioid substitution therapy
PHQ-9: Patient Health Questionnaire-9
PIEs: Psychologically informed environments
PIP: Personal independence payment
PN/s: Peer Navigator/s
RAND SF-36: RAND Short-Form-36
RCT: Randomised Controlled trial
SHARPS: Supporting Harm Reduction through Peer Support
SD: Standard deviation
SDF: Scottish Drugs Forum
SURE: Substance Use Recovery Evaluator
TSA: The Salvation Army
UK: United Kingdom
