# Supplementary material for: Assessing the feasibility, acceptability and accessibility of a peer-delivered intervention to reduce harm and improve the well-being of people who experience homelessness with problem substance use: the SHARPS study
Source: Harm Reduct J. 2022 Feb 4;19:10. doi: 10.1186/s12954-021-00582-5 (PMC8815224; doi:10.1186/s12954-021-00582-5)
Supplement: Supplementary file 1 — Additional file 1. Application of Normalisation Process Theory for the assessment of feasibility, acceptability and accessibility. [file 12954_2021_582_MOESM1_ESM.docx]

**Additional file 1: Application of Normalisation Process Theory to the assessment of feasibility, acceptability and accessibility**

| **Coherence** | This related to the value placed on the Peer Navigators’ role by themselves and others, particularly participants and staff in intervention settings. In order to embrace the role, those involved needed to be clear about, and fully understand, the rationale for the intervention, as well as its core values (e.g. PIEs, harm reduction). The Peer Navigators benefitted from a lengthy induction period which enabled them to understand their role and how it would fit within their service. Prior to the start of the study, service managers and organisational leaders were involved in a number of discussions about the role and were involved throughout the study on key decisions and progress. All had a high level of understanding of the study and its values. While service staff (e.g. Support Workers) were given information about the study, findings indicate that they would have benefitted from receiving more information, at an earlier stage. This would have helped the intervention to have been better understood and valued by all and made it easier for the Peer Navigators to become better embedded in the settings at an earlier stage. |
| --- | --- |
| **Cognitive participation** | To ensure this intervention could be implemented effectively, support or ‘buy-in’ was required from a number of individuals within a range of organisations. Participants witnessed the Peer Navigators’ practice in their services, were quickly receptive to the Peer Navigators, and were able to get to know them over time. Service managers helped the Peer Navigators to settle into their service and respective organisations, and supported them throughout the study through joint line management and informal support. Other members of staff supported the intervention within their own roles, for example, the administration of the participant support monies fund, referring service users/residents to the Peer Navigators, and signposting the Peer Navigators to local contacts. Participants, the Peer Navigators, and staff in the intervention settings generally ‘bought in’ to the intervention, and became more supportive of the intervention as it developed. Greater understanding of the intervention would likely have facilitated greater buy-in. |
| **Collective action** | To ensure the intervention could be implemented, service staff made a number of adjustments to their work practice. For example, staff made workspaces available for the Peer Navigators as far as possible (though these were affected by the limited space within some of the services) and made introductions to other individuals/organisations to support the Peer Navigators in their roles. Over time, staff also adjusted to the different role the Peer Navigators had, compared to themselves and others in their service, although this sometimes manifested as tensions, connected to a lack of understanding of the role and also, we believe, due to stigma towards people who have overt histories of problem substance use. The Peer Navigators also adjusted to the working practices of their setting and understood the importance of communication with all staff. |
| **Reflexive monitoring** | The Peer Navigators, and all staff in the intervention settings, continually appraised how the intervention was going in their respective service though informal communication, formal meetings, group reflective practice, within their staff teams and with the study team. The Peer Navigators’ role evolved during the course of the study. Each had their own ways of working but they also made adjustments as needed to suit their individual participants and the context in which they worked. Interviews conducted with participants, staff, and Peer Navigators over the course of the study, reflective diary keeping, and regular note-taking by the project management team ensured that the feasibility, accessibility and acceptability of the intervention was being continually assessed and responded to by the study team, with adjustments being made as necessary. An example here included having regular conversations with the Peer Navigators about the importance of record keeping. |
